# Source of SARS-CoV-2 infection: results from a series of 584,846 cases in France from October 2020 to August 2022

**DOI:** 10.1186/s12889-024-17772-y

**Published:** 2024-01-30

**Authors:** Arthur Rakover, Simon Galmiche, Tiffany Charmet, Olivia Chény, Faïza Omar, Christophe David, Sophie Martin, Alexandra Mailles, Arnaud Fontanet

**Affiliations:** 1 Emerging Diseases Epidemiology Unit, Institut Pasteur, Université Paris Cité, 25 Rue du Docteur Roux, 75015 Paris, France; 2 Sorbonne Université, Ecole Doctorale Pierre Louis de Santé Publique, Paris, France; 3 Institut Pasteur, Université Paris Cité, Centre for Translational Research, Paris, France; 4 Institut Ipsos, Paris, France; 5 Caisse Nationale de L’Assurance Maladie, Paris, France; 6 Santé Publique France, Saint-Maurice, France; 7 Conservatoire National Des Arts Et Métiers, Unité PACRI, Paris, France

**Keywords:** COVID-19, SARS-CoV-2, Transmission, Household

## Abstract

**Background:**

We aimed to study the source of infection for recently SARS-CoV-2-infected individuals from October 2020 to August 2022 in France.

**Methods:**

Participants from the nationwide ComCor case–control study who reported recent SARS-CoV-2 infection were asked to document the source and circumstances of their infection through an online questionnaire. Multivariable logistic regression was used to identify the factors associated with not identifying any source of infection.

**Results:**

Among 584,846 adults with a recent SARS-CoV-2 infection in France, 46.9% identified the source of infection and an additional 22.6% suspected an event during which they might have become infected. Known and suspected sources of infection were household members (30.8%), extended family (15.6%), work colleagues (15.0%), friends (11.0%), and possibly multiple/other sources (27.6%). When the source of infection was known, was not a household member, and involved a unique contact (*n* = 69,788), characteristics associated with transmission events were indoors settings (91.6%), prolonged (> 15 min) encounters (50.5%), symptomatic source case (64.9%), and neither the source of infection nor the participant wearing a mask (82.2%). Male gender, older age, lower education, living alone, using public transportation, attending places of public recreation (bars, restaurants, nightclubs), public gatherings, and cultural events, and practicing indoor sports were all independently associated with not knowing the source of infection.

**Conclusion:**

Two-thirds of infections were attributed to interactions with close relatives, friends, or work colleagues. Extra-household indoor encounters without masks were commonly reported and represented avoidable circumstances of infection.

**Trial registration:**

ClinicalTrials.gov registration number: NCT04607941.

**Supplementary Information:**

The online version contains supplementary material available at 10.1186/s12889-024-17772-y.

## Introduction

The airborne spread of SARS-CoV-2 [1, 2] and the possibility of transmission by asymptomatic individuals [3] have considerably broadened the range of opportunities for viral transmission relative to pathogens for which close contact with symptomatic patients are required. Places where the risk is highest include poorly ventilated indoor environments where people do not systematically wear masks [4]. Several types of public places (bars, restaurants, indoor sports) and means of transportation (long-distance buses, airplanes) have been shown to be associated with an increased risk of transmission [5–8], with large clusters when people speak loudly and sing [9, 10]. As a result, the control of viral circulation has relied on the wearing of masks and the closure of public places, transportation, schools, and workplaces, and the implementation of contact tracing in May 2020 in France. This strategy has also involved imposing curfews and confinements, with major social and economic repercussions. However, the relative contribution of private and public places in overall transmission and that of family, friends, and work colleagues as sources of infection has been little studied. It is also unknown whether the relative contribution varied depending on the circulating variants and the nature of countermeasures applied during pandemic waves. This information may be relevant for the targeting of prevention and control measures for this and future airborne-transmitted epidemics. In this study, we present findings from a nationwide dataset analysis in France that includes information on 584,846 adults who were infected with SARS-CoV-2 between October 2020 and August 2022. Our objective was to study the circumstances of infection in recently SARS-CoV-2-infected individuals.

## Methods

### Participants and data collection

For this project, we used data from the ComCor case–control study, which we have previously described [7, 11, 12]. Adults with acute SARS-CoV-2 infection confirmed by RT-PCR or a rapid antigen test (except self-administered antigen tests) were invited to participate by email by the *Caisse Nationale d'Assurance Maladie* – the national health insurance agency, which receives notification of all SARS-CoV-2 infections in France. Cases received information online about the study before completing a questionnaire if they agreed to participate. For the present study, we included cases diagnosed between October 1, 2020, and August 29, 2022. Healthcare workers were excluded from the present analysis, as they were assumed to have higher occupation-related exposure to SARS-CoV-2 than the general population [13–15].

The questionnaire covered sociodemographic characteristics (age, sex, region, population of the area of residence, profession, and level of education) and comorbidities, as well as a series of potential situations of exposure in the days preceding infection from inside and outside the household (work habits, modes of transportation, attendance at restaurants and bars, etc.). We also collected information on the presumed source of infection (hereafter designated as case source), when identified by the participant, or the suspicion of a particular event during which infection likely occurred (hereafter designated as suspected event). In early 2022, based on the results of a qualitative study showing that half of participants who had answered “no known source” had, in fact, multiple suspected sources without being sure which one was involved, we introduced the option to report the most likely source while mentioning it was uncertain.

The questionnaire focused on the 10 days preceding symptom onset (or test for asymptomatic participants). This period was reduced to seven days in January 2022 following emergence of the B.1.1.529 (Omicron) variant, given its shorter incubation period [16].

The study was divided into nine periods according to the epidemic waves, emergence of variants, and non-pharmaceutical interventions, including lockdowns or curfews, as described in Figure S1. Major changes in restrictions throughout the study period included national lockdowns from October 30, 2020, to November 28, 2020, and from April 3, 2021, to May 19, 2021, as well as a nationwide curfew implemented on December 15, 2020, and lifted on June 20, 2021.

### Statistical analysis

We recorded the sociodemographic characteristics, sources of SARS-CoV-2 infection, and behaviours before and during SARS-CoV-2 infection for all participants infected between October 1, 2020, and August 29, 2022. In addition, we analyzed these characteristics over nine different time periods.

To better characterize cases without an identified source of infection, we used multivariable logistic regression to identify the sociodemographic factors and behavioral exposure associated with the profile. Variables introduced into the models were age (in ten-year categories), gender, region, population of the area of residence, and calendar week, as well as level of education, professional category, comorbidities (diabetes mellitus, hypertension, chronic respiratory disease, body-mass index), smoking status, living alone, vaccination status (measured in number of doses), past history of SARS-CoV-2 infection, exposure in the days before infection (use of public transportation, private and public gatherings, cultural events, indoor sports, bars, restaurants, nightclubs, shops), and prevention measures (mask-wearing, hand-washing, physical distancing). We inspected the correlation between different community exposures before using them as covariates**.**

All statistical analyses were performed using Stata 16.0 (StataCorp, College Station, TX, USA).

## Results

### Sociodemographic characteristics

From October 27, 2020, to August 29, 2022, 11,446,403 adult individuals with a diagnosis of SARS-CoV-2 infection were contacted by e-mail by the national insurance program, of whom 680,396 (5.9%) replied. After the exclusion of healthcare workers (*n* = 83,919) and participants with inconsistencies regarding the region of residence (French overseas territories while these were not targeted by the email invitations, missing region), the circumstances of transmission (e.g., details provided for workplace transmission while previously reporting intra-familiar transmission), or a past episode of infection (episode < 2 months prior to the ongoing episode),, 584,846 participants with SARS-CoV-2 infection were retained for analysis. Participant characteristics are described in Table S1. Compared to the national SARS-CoV-2 infection database (système d’information de dépistage—SI-DEP—data available between October 1, 2020, and March 12, 2022) [17], our study population was more predominantly female (66.0% versus 53.6% in the SI-DEP), and older (66.1% older than 40 versus 56.2% in the SI-DEP), except for the oldest age group of 70 years and older.In addition, relative to the French general population (data provided by the Institute of National Statistics and Economic Studies, INSEE), our study population had a higher socioeconomic status (31.1% were senior executives versus 21.6% in the INSEE database) [18]. The main characteristics remained largely stable throughout the study, except for a higher share of participants aged > 50 years during the last two periods (March 18 to August 29, 2022) compared to before (October 1, 2020, to March 17, 2022) (51.9% vs 32.8%, respectively, *P* < 0.001; Table S2).

### Source of SARS-CoV-2 infection

**Fig. 1:**
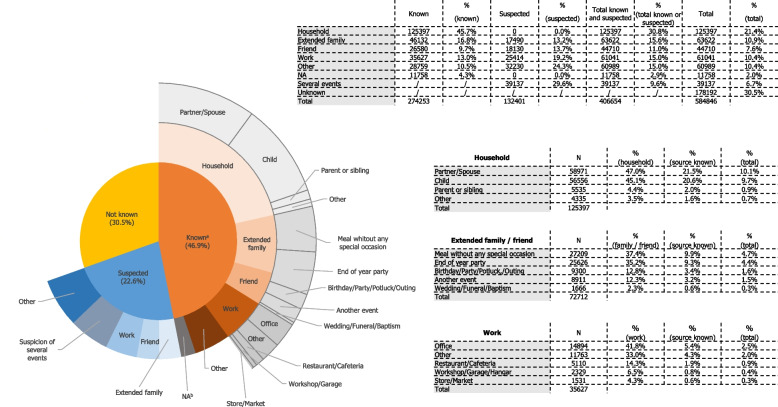
Characteristics of the source cases of SARS-CoV-2 infection from inside and outside the household. **a** Source known: 9.1% expressed doubts on the identification of the source case (option introduced in the questionnaire in January 2022); SARS-CoV-2 infection was confirmed with a test in 88% of the source cases. **b** Not applicable: 11,758 participants were considered to have missing values; between January 6 and March 1, 2022, participants who expressed doubts about the source of contamination were unable to provide details

Approximately two-thirds (69.5%) of the participants knew the source of their SARS-CoV-2 infection or suspected one or more events related to the infection. More specifically, 46.9% were able to identify a source case (confirmed by a positive SARS-CoV-2 tests for 88.0% of them), 22.6% suspected one or more specific events during which transmission might have occurred, and 30.5% did not know how they were infected (see Fig. 1). Household members, extended family, colleagues, and friends represented 45.7%, 16.8%, 13.0%, and 9.7% of the sources of infection, respectively, when known; 30.8%, 15.6%, 15.0%, and 11.0% of the sources of infection, respectively, when known or suspected; and 21.4%, 10.9%, 10.4%, and 7.6% of all infections, respectively (see Fig. 1). The characteristics of the source cases reported by the participants are presented in Table S3.

**Fig. 2:**
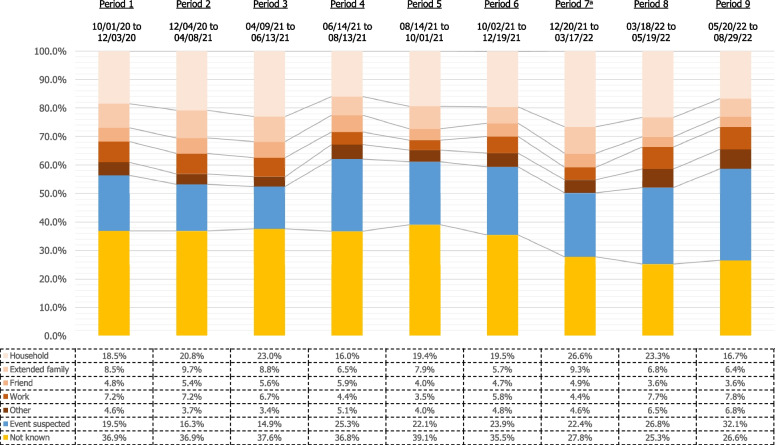
Characteristics of the sources of SARS-CoV-2 infection from inside and outside the household by period. **a** From January 2022 on, it was possible for participants to mention whether they had doubts or not about the identification of the source of infection

These proportions remained somewhat stable throughout the study period, except for the proportion of unknown sources, which decreased from a range of 35% to 39% during the first six periods (October 1, 2020 to December 19, 2021) down to 25% to 28% during the last three periods (December 20, 2021 to August 19, 2022) when the option of identifying a source of infection “with some doubt” was offered. We also observed an increase in the proportion of suspected events and a decrease in household or workplace infections between periods 1 to 3 (characterized by high stringency of non-pharmaceutical interventions) and periods 4 to 6, during which most restrictions were lifted and social interactions likely increased, making the correct identification of sources potentially more difficult (see Fig. 2).

**Fig. 3:**
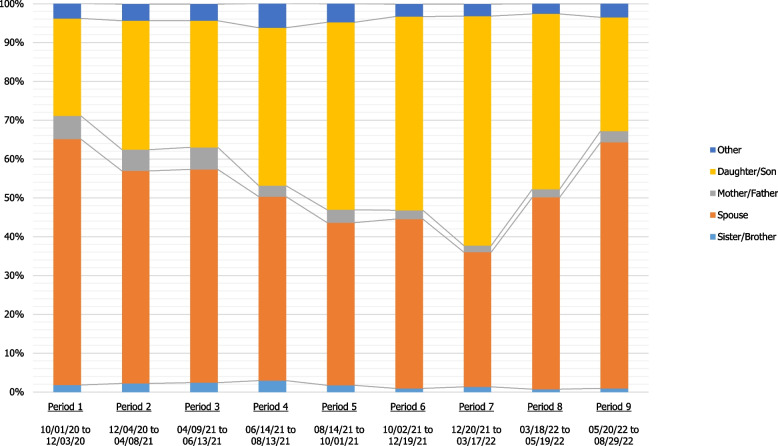
Distribution of the relationship to source cases of SARS-CoV-2 infection in the household by period

When transmission occurred at home (45.7% of those with an identified source of infection), the source of infection was most frequently a partner/spouse (47.0%), followed by a child (45.1%), and the source of infection was most often (87.3%) symptomatic. The proportion of children as a household source of infection increased from 25.1% during the first period (October 1 to December 3, 2020) to 58.1% during the seventh period (December 20, 2021 to March 17, 2022), before decreasing to 29.3% during the last period (May 20, 2022 to August 29, 2022) (see Fig. 3).

When transmission occurred outside of the household (54.3% of those with an identified source of infection) and involved extended family and friends (48.9% of extra-household sources of infection), transmission occurred mostly during meals (37.4%), end-of-the-year celebrations (mainly Christmas and New Year’s Eve) (35.2%), birthday parties or potluck outings (12.8%), and family ceremonies, such as weddings, funerals, and baptisms (2.3%) (see Fig. 1). When transmission involved colleagues (23.9% of identified extra-household sources of infection), transmission took place primarily in shared offices (41.8%), followed by restaurants/cafeterias (14.3%).

**Fig. 4:**
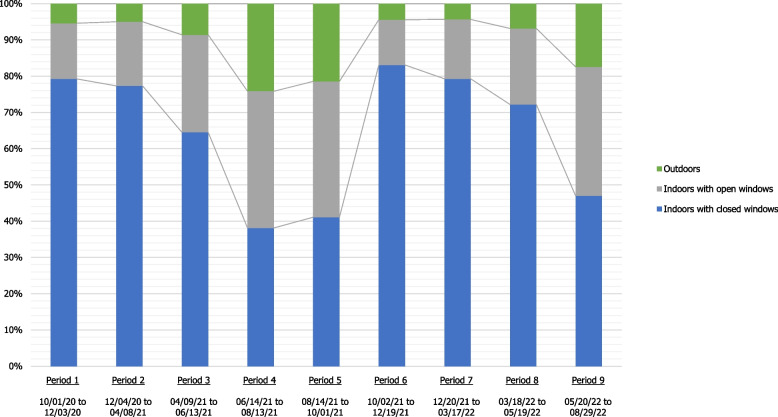
Location of single encounters resulting in SARS-CoV-2 transmission among participants with an identified source by period

A single interaction with the source case was reported for half (50.9%) of the extra-household transmission events with a known source of infection. Source cases were symptomatic in 35.1% of these encounters, varying according to whether they were extended family, friends, or work colleagues (38.7%, 27.9%, and 44.1%, respectively, *P* < 0.001) and the time period (Figure S2). An average half of infections took place during prolonged (over 15 min) encounters (50.5%) (but more than 20% during an encounter shorter than 5 min) and in indoor settings (91.6%) (Table 1). Seasonality influenced the setting in which transmission occurred, with indoor spaces with closed windows accounting for approximately 80% of transmission during winter periods and approximately 40% during summer periods (see Fig. 4). The proportion of encounters that caused an infection during which neither the source case nor the participant wore a mask remained high and stable for encounters with family and friends (91.2% and 95.6%, respectively) and increased from 46.6% during the first period (October 1, 2020 to December 3, 2020) to 85.4% during the last period (May 20, 2022 to August 29, 2022) for encounters at work (Figure S3). Furthermore, the characteristics of the encounters (location, duration, mask-wearing) appeared to be minimally affected by the symptomatic status of the source case, except for mask-wearing (Table S4). Individuals who suspected an event but were unable to identify a source case, who accounted for 22.6% of all cases, reported shared meals (18.2%) and meetings (10.5%) as the most common type of event (Table S5). These events primarily took place in work (27.2%), family (18.8%), or friendly (19.4%) settings.

**Table 1:** Characteristics of single-encounter meetings during which SARS-CoV-2 infection took place according to the origin of the source case (household members excluded) between October 1, 2020, and August 29, 2022

	**Total (*n* = 69,788)**		**Work (*n* = 12,164)**		**Family (*n* = 21,668)**		**Friends (*n* = 18,164)**		**Other**^**a**^** (*n* = 17,784)**		***P* value**
	N	%	N	%	N	%	N	%	N	%	
Symptomatic											< *0.001*
Yes	24,529	35.1%	5366	44.1%	8379	38.7%	5070	27.9%	5712	32.1%	
Location of encounter											
Indoors with closed windows	49,230	70.6%	9323	76.6%	15,323	70.7%	12,512	68.9%	12,072	67.9%	
Indoors with open windows	14,687	21.0%	2259	18.6%	5081	23.4%	4211	23.2%	3136	17 0.6%	
Outdoors	5863	8.4%	582	4.8%	1264	5.8%	1441	7.9%	2576	14.5%	
Duration of encounter											< *0.001*
< 1 min	6350	9.1%	1528	12.6%	1885	8.7%	1389	7.6%	1548	8.7%	
< 5 min	8688	12.5%	2073	17.0%	2463	11.4%	1849	10.2%	2303	12.9%	
5–15 min	9594	13.7%	2140	17.6%	2681	12.4%	2049	11.3%	2724	15.3%	
> 15 min	35,206	50.5%	4809	39.5%	10,978	50.7%	10,430	57.4%	8988	50.5%	
Unknown	9943	14.2%	1614	13.3%	3661	16.9%	2447	13.5%	2221	12.5%	
Mask-wearing^b^											< *0.001*
Neither wore a mask	57,330	82.2%	7169	58.9%	19,751	91.2%	17,364	95.6%	13,046	73.4%	
By suspected source of infection only	2088	3.0%	543	4.5%	598	2.7%	227	1.2%	720	4.1%	
By person infected only	4223	6.0%	1572	12.9%	601	2.8%	272	1.5%	1778	10.0%	
By both source case and participant	6139	8.8%	2880	23.7%	718	3.3%	301	1.7%	2240	12.6%	

^a^“Other" refers to any contamination that occurs in a cultural, sporting, religious, or health-related environment

^b^Mask-wearing during the last encounter with the source when multiple encounters occurred

Approximately 30% of all participants did not know how they were infected. In the multivariable logistic regression model, the factors independently associated with not knowing the source or event responsible for the infection were male gender, older people, low level of education, living alone, use of short-distance transport (bus, tram, subway, and train), or national or international transport (airplane, train, bus, cruise ship), frequentation of bars or restaurants, cultural venues (theatre, cinema, museum, concert, festival), retail or shops, public gatherings (school or university and religious), and indoor sports. When we analyzed nightclubs and private parties separately in a separate model with fewer observations (the initial questionnaire did not differentiate between these different types of parties), we found that nightclubs were associated with a higher risk of an unknown source of infection. (Table 2) We observed only limited correlation between different exposures of interest (transport, gatherings, sports, parties, bars and restaurants) with correlation coefficient below 0.5. Thus we included all these variables as covariates in the regression model**.**

**Table 2:** Factors associated with not knowing the source of contamination in logistic regression analysis

	***N* (%) Source: known or suspected**^**a**^** (*n* = 273,497)**	***N* (%) Source: not known**^**a**^** (*n* = 309,334)**	**Univariable analysis OR (95% CI)**	**Multivariable analysis Adjusted OR (95% CI)**^**b**^
Gender				
Female	188,369 (68.9)	196,638 (63.6)	1 (ref)	1 (ref)
Male	85,128 (31.1)	112,696 (36.4)	1.27 (1.25–1.28)	1.19 (1.18–1.21)
Age (years)				
18 – 29	37,107 (13.6)	42,083 (13.6)	1 (ref)	1 (ref)
30 – 39	67,105 (24.5)	65,969 (21.3)	0.87 (0.85–0.88)	1.05 (1.03–1.07)
40 – 49	73,814 (27.0)	73,798 (23.9)	0.88 (0.87–0.90)	1.09 (1.07–1.09)
50 – 59	52,519 (19.2)	66,118 (21.4)	1.10 (1.09–1.13)	1.24 (1.21–1.26)
60 – 69	30,819 (11.3)	41,368 (13.4)	1.18 (1.16–1.21)	1.27 (1.24–1.30)
≥ 70	12,133 (4.4)	19,998 (6.5)	1.45 (1.41–1.50)	1.53 (1.49–1.60)
Level of education				
No high school diploma	42,035 (15.4)	57,066 (18.4)	1 (ref)	1 (ref)
High school diploma	49,528 (18.1)	56,249 (18.2)	0.84 (0.82–0.85)	0.85 (0.83–0.86)
Bachelor's degree	95,733 (35.0)	99,859 (32.3)	0.77 (0.76–0.78)	0.75 (0.74–0.76)
Master's degree	86,201 (31.5)	96,160 (31.1)	0.82 (0.81–0.83)	0.68 (0.67–0.69)
Living alone	38,489 (14.1)	68,623 (22.2)	1.74 (1.71–1.76)	1.50 (1.48–1.53)
Short-distance transportation				
Bus	23,493 (8.6)	38,932 (12.6)	1.53 (1.51–1.56)	1.16 (1.14–1.19)
Tram	12,918 (4.7)	20,155 (6.5)	1.41 (1.37–1.44)	1.10 (1.07–1.13)
Subway	28,427 (10.4)	47,331 (15.3)	1.56 (1.53–1.58)	1.18 (1.16–1.21)
Train	13,759 (5.0)	21,319 (6.9)	1.40 (1.37–1.43)	1.08 (1.06–1.11)
Car-pooling				
Yes	57,040 (20.8)	71,057 (30.0)	1.13 (1.11–1.15)	1.00 (0.99–1.02)
Car-pooling with relatives^c^	43,631 (15.9)	54,780 (17.7)	1.14 (1.13–1.15)	0.99 (0.97–1.00)
Car-pooling booked on a platform^c^	1775 (0.6)	2713 (0.9)	1.37 (1.29–1.46)	1.06 (1.00–1.13)
Long-distance national or international travel				
Airplane	5461 (2.0)	12,754 (4.1)	2.11 (2.04–2.18)	1.60 (1.55–1.66)
Train	10,971 (4.0)	18,408 (6.0)	1.51 (1.46–1.55)	1.11 (1.07–1.14)
Bus	2571 (0.9)	5659 (1.8)	1.95 (1.86–2.05)	1.11 (1.05–1.17)
Cruise ship	657 (0.2)	1559 (0.5)	2.06 (1.88–2.59)	1.14 (1.04–1.26)
Private gathering				
Ceremony (marriage, Funeral, etc.)	4251 (1.6)	5679 (1.8)	1.18 (1.14–1.23)	1.06 (1.02–1.11)
Meal, without special occasion	55,675 (20.4)	62,360 (20.2)	0.99 (0.97–1.00)	0.90 (0.89–0.92)
Coffee	24,836 (9.1)	29,709 (9.6)	1.06 (1.04–1.08)	0.97 (0.95–0.99)
Birthday	20,224 (7.4)	22,827 (7.4)	1.00 (0.98–1.02)	0.94 (0.92–0.96)
Family or friendly party	31,579 (11.5)	32,427 (10.5)	0.90 (0.88–0.91)	0.87 (0.86–0.89)
Public gathering				
School or university	17,105 (6.3)	21,351 (6.9)	1.19 (1.16–1.23)	1.21 (1.18–1.23)
Religious	9124 (3.3)	12,250 (4.0)	1.11 (1.09–1.13)	1.09 (1.06–1.13)
Retail, shops	186,598 (45.2)	226,128 (54.8)	1.26 (1.25–1.28)	1.19 (1.17–1.20)
Cultural events				
Yes	28,946 (10.6)	48,961 (15.8)	1.59 (1.56–1.61)	1.33 (1.31–1.36)
Theatre^d^	3873 (1.4)	6301 (2.0)	1.45 (1.39–1.51)	1.13 (1.08–1.18)
Cinema^d^	11,851 (4.4)	16,928 (5.5)	1.28 (1.25–1.31)	1.07 (1.05–1.10)
Museum^d^	3576 (1.3)	7132 (2.3)	1.78 (1.71–1.86)	1.14 (1.09–1.19)
Concert^d^	4274 (1.6)	8718 (2.8)	1.83 (1.76–1.89)	1.50 (1.44–1.56)
Festival^d^	3191 (1.6)	6814 (3.0)	1.94 (1.86–2.03)	1.52 (1.45–1.58)
Indoor sports	16,149 (5.9)	22,168 (7.2)	1.23 (1.20–1.26)	1.17 (1.14–1.19)
Bar or restaurants	72,623 (26.6)	110,510 (35.7)	1.54 (1.52–1.55)	1.39 (1.37–1.41)
Nightclub or private parties				
Yes	26,284 (9.6)	33,732 (10.9)	1.15 (1.13–1.17)	0.98 (0.97–1.01)
Nightclub^e^	3188 (1.6)	8165 (3.7)	2.35 (2.26–2.45)	1.56 (1.49–1.63)
Privates parties^e^	27,062 (12.2)	27,062 (12.2)	1.06 (1.04–1.07)	0.89 (0.87–0.91)

^a^2015 participants were excluded from the model due to missing data, resulting in a total effective sample size of 582,831

^b^Multivariable model adjusted for all variables shown in the model, as well as region of residency, population density of the place of residence, comorbidities (diabetes mellitus, hypertension, coronary artery disease, chronic respiratory disease,), body-mass index, smoking status, calendar week, housing type, history of COVID-19 and prevention measures (mask-wearing, hand-washing, physical distancing)

^c^The questionnaire was modified on June 29, 2021 to obtain detailed information on the type of car-pooling (with relatives or on a platform). Odds ratios for other variables are those estimated in a model containing car-pooling as a binary variable so that all observations could be kept in the model. Odds ratios for specific cultural events were estimated in a distinct multivariable model on 452,631 observations with information on the type of car-pooling (results for other estimates are not shown here)

^d^The questionnaire was modified on December 16, 2020, to obtain detailed information on the type of cultural event. Odds ratios for other variables are those estimated in a model containing cultural events as a binary variable so that all observations could be kept in the model. Odds ratios for specific cultural events were estimated in a distinct multivariable model on 580,025 observations with information on the type of cultural event (results for other estimates are not shown here)

^e^The questionnaire was modified on July 28, 2021, to detail the type of party. Odds ratios for other variables are those estimated in a model containing parties as a binary variable so that all observations could be kept in the model. Odds ratios for a specific party type (nightclub or private party) were estimated in a distinct multivariable model on 422,698 observations with information on the type of party (results for other estimates are not shown here)

## Discussion

We present a descriptive analysis of the circumstances of contamination of 584,846 adults with a recent SARS-CoV-2 infection in France. Approximately two-thirds of cases believed they knew how they were infected, either because they were able to identify the source of infection (46.9%), or a specific event was suspected (22.6%). When the source of infection was known, household members were the most frequent source (45.7%), followed by extended family (16.8%), workplace (13.0%), and friends (9.7%). When the source of infection was unknown (30.5% of all cases), participants were more likely to have visited places involving high rates of contacts with unrelated or unknown people, such as places of public recreation or public transportation, than other participants. The beginning of the study was characterized by stringent non-pharmaceutical interventions (closures, curfews, and lockdowns), which translated into fewer suspicious events and more infections at home compared to periods when restrictions were eased. Social interactions likely increased, making the identification of potential sources more complex. However, the distribution of settings of transmission were overall stable, with the household remaining the main setting of known transmissions.

Households were the main drivers of infection, representing approximately 45.7% of all infections with a known source and at least 21.4% of all infections (30.5% of all infections were of unknown origin and some may have been due to household transmission). Several previous studies have shown the high transmissibility of SARS-CoV-2 in household settings [19–21]. Sun et al. found that the risk of transmission was highest among household contacts, followed by extended family, social, and community contacts [22]. A systematic review and meta-analysis of household transmission of SARS-CoV-2 found an overall secondary attack rate (SAR) of 18.9% (95% CI, 16.2%-22.0%), with a higher estimate for the omicron variant (42.7%, 95% CI 35.4%-50.4%) [23]. Infection in the household for our adult participants was predominantly from the spouse or partner at the beginning of the pandemic, but we noted an increase in the proportion of infections by children, who became the most frequent source of household contamination during the omicron BA.1 wave. Thus The emergence of more transmissible variants (first delta and then omicron) in a population with relatively low pre-existing immunity, combined with the lifting of health restrictions and the progress of the adult vaccination campaign, is likely to have encouraged more efficient circulation in these age groups from summer 2021 [17, 24]. The following decrease in the proportion of infection by children in the last period of the study may reflect transient herd immunity in children following the intense circulation of the Omicron BA.1 variant in the winter of 2022.

Private gatherings with family or friends were the second most common circumstance of infection in our study population. They represented 26.5% of infections with a known source and at least 18.5% of all infections. Contacts at social events with family and friends have been shown to be associated with a higher risk of transmission than other low-risk casual contacts, with a documented SAR of 5.9% [20]. These gatherings often included meals and masks were not worn in more than 90% of single encounters that resulted in transmission (Figure S3). End-of-the-year celebrations were also reported as important sources of transmission, particularly at the end of 2021, when they occurred during the omicron BA.1 wave and incidence rates were particularly high.

The workplace was the third most common location associated with transmission, representing 13.0% of infections with a known source and at least 10.4% of all infections. Offices and cafeterias were the locations associated with most transmission events when the source was known. It is noteworthy that the proportion of encounters without masks associated with transmission at work increased from 40 to 50% in 2021 to 70% in the spring of 2022 and 85% in the summer of 2022 (mask mandates at work ended on March 12, 2022).

We were able to obtain information on a large number (> 60,000) of single encounters that resulted in transmission. It suggests that prolonged (> 15 min) interactions without masks in indoor settings resulted in the largest number of infections, consistent with the existing literature (19,20), although it's observed that that brief encounters (< 5 min) still contribute to 20% of infections, and this proportion increases even more within a workplace context. However, up to 20% of transmission events still took place outdoors during the summer months, although misclassification of the setting or infecting source cannot be excluded in some cases. In 35% of such encounters, the source of infection was symptomatic. Conversely, this figure suggests that two thirds of transmission events were caused by individuals who were asymptomatic at the time of transmission, whether they were in the pre-symptomatic phase or remained asymptomatic for the duration of the infection. This figure is consistent with those of other published articles, which highlight a significant proportion of infections among asymptomatic individuals [25–28], but should be considered with caution, as symptoms in the source case may have been missed by the index case, this could be attributed to either mild symptoms or difficulties in accurately identifying them. It is nevertheless troubling to realize that many transmission events took place involving infected individuals who were symptomatic at the time of transmission and unmasked, some in the work setting.

Our findings are subject to some limitations due to the study design. The very large number of cases available for analysis should not hide the low (5.9%) response rate to the online questionnaire. The study population was more highly female, aged 30 to 49 years, with few people aged 70 and above (possibly due to the study being conducted online) and had more post-secondary education than the group registered in the SI-DEP cases database, thus suggesting the possibility of a selection bias and potentially affecting the generalizability of the findings [29]. We relied on the interpretation of the transmission chain by the participants. It is plausible that some interpretations were erroneous, given possible multiple sources of exposure, particularly during the Omicron waves in 2022, when the incidence was very high. We were unable to further validate the correct identification of the source case. It is, however, reassuring that participants reported a positive SARS-CoV-2 test (PCR or rapid antigen test) for 88.0% of the source cases that infected them. Symptomatic status of the source case was not a requirement for the identification of the transmission: transmission from asymptomatic sources could be identified for instance if the source case developed symptoms and tested positive little after the contact resulting in transmission. This explains why we identified close to 64.8% of asymptomatic sources when transmission happened during single encounters. It is also expected that the description of the circumstances of infection may, at least partially, reflect the knowledge and beliefs of the participants on the determinants of SARS-CoV-2 transmission. Thus, we may have missed some transmission events that occurred in circumstances generally recognized as unusual (eg, outdoors, during brief encounters) and that would not have been identified by the participants.

Few studies have used a similar approach to estimate the relative contribution of different settings to the sources of SARS-CoV-2 transmission. Vaux et al. and Thompson et al. have also identified the predominant role of the household and the workplace [20, 30]. However, our approach, involving the evaluation of different phases of the epidemic based on criteria such as epidemic waves, the presence of variants, and control measures, enhances the understanding of contamination circumstances. This study provides novel insights, notably the observation that half of the individuals infected outside the household contracted the virus during a single encounter. Moreover, it allows for the introduction of nuanced perspectives, particularly regarding the role of enclosed spaces during summer periods.

This study provides a picture, albeit imperfect, of the most relevant settings that public health strategies should target to mitigate transmission of SARS-CoV-2, namely households, as well as private gatherings with family or friends and the workplace. This is important, as it is possible that with aerosol transmission, the number of places at risk might have increased to the point that transmission would no longer be traceable. These results complement previous findings from the same study, which identified an increased risk of SARS-CoV-2 infection associated with an increasing number of household members, the attendance of bars or restaurants, and professional meetings, amongst others [7, 12].

Our study offers valuable insights into the circumstances of SARS-CoV-2 infection and, as such, should help guide public health policies aimed at mitigating SARS-CoV-2 transmission. By understanding these factors, public health policies can be tailored to address the identified sources of transmission. This could involve focused interventions, such as advocating specific hygiene practices, implementing social distancing measures in particular settings, or enhancing air quality in specific locations like workplaces. Moreover, our results also provide data for improving pandemic preparedness strategies.

### Supplementary Information

**Additional file 1. **

## Data Availability

The data that support the findings of this study are available at Institut Pasteur. Restrictions apply to the availability of these data, which were used under authorized agreement for this study from the data protection authority, the Commission Nationale de l’Informatique et des Libertés (CNIL). Access to these data would therefore require prior authorization by the CNIL. Contact the corresponding author (Arnaud Fontanet, arnaud.fontanet@pasteur.fr) for requests.
