# Supplementary material for: Source of SARS-CoV-2 infection: results from a series of 584,846 cases in France from October 2020 to August 2022
Source: BMC Public Health. 2024 Jan 30;24:325. doi: 10.1186/s12889-024-17772-y (PMC10826227; doi:10.1186/s12889-024-17772-y)

Table S1. Characteristics of 584,846 cases of SARS-CoV-2 infection between October 1, 2020, and August 29, 2022

|  | N | (%) |
| --- | --- | --- |
| Age (years) |  |  |
| 18 – 29 | 69336 | 11.9% |
| 30 – 39 | 128556 | 22.0% |
| 40 – 49 | 148082 | 25.3% |
| 50 – 59 | 124130 | 21.2% |
| 60 – 69 | 77224 | 13.2% |
| ≥ 70 | 37518 | 6.4% |
| Gender |  |  |
| Female | 386182 | 66.0% |
| Region of residence |  |  |
| Île-de-France | 126500 | 21.6% |
| Auvergne-Rhône-Alpes | 83712 | 14.3% |
| Occitanie | 53889 | 9.2% |
| Grand Est | 50890 | 8.7% |
| Provence-Alpes-Côte d'Azur and Corsica | 50510 | 8.6% |
| Nouvelle-Aquitaine | 48012 | 8.2% |
| Hauts-de-France | 48001 | 8.2% |
| Pays de la Loire | 31224 | 5.3% |
| Bretagne | 25751 | 4.4% |
| Bourgogne-Franche-Comté | 23578 | 4.0% |
| Normandie | 23094 | 3.9% |
| Centre - Val de Loire | 19685 | 3.4% |
| Population of place of residence |  |  |
| Rural or < 5,000 | 146230 | 25.0% |
| 5,000 – 19,999 | 59244 | 10.1% |
| 20,000 – 99,999 | 69280 | 11.8% |
| 100,000 + | 195997 | 33.5% |
| Paris urban area | 114095 | 19.5% |
| Professional category |  |  |
| Senior executives | 176995 | 31.1% |
| Intermediate profession | 97984 | 17.2% |
| Employees | 117577 | 20.6% |
| Worker/Farmer/ Independent Profession. | 50662 | 8.9% |
| Retired | 81718 | 14.4% |
| Unemployed or inactive | 23826 | 4.2% |
| Student | 20876 | 3.7% |
| Level of education |  |  |
| No high school diploma | 99717 | 17.1% |
| High school diploma | 106138 | 18.1% |
| Bachelor's degree | 195992 | 33.5% |
| Master's degree | 182999 | 31.3% |
| Living alone |  |  |
| Yes | 107535 | 18.4% |
| House | 354835 | 60.7% |
| Apartment | 227422 | 38.9% |
| Shelters and nursing homes | 2589 | 0.4% |
| [Comorbidities](https://context.reverso.net/traduction/anglais-francais/comorbidities) |  |  |
| Obesity | 92862 | 15.9% |
| Hypertension | 59645 | 10.2% |
| Chronic respiratory disease | 48476 | 8.3% |
| Immunosuppression | 22499 | 4.0% |
| Diabetes mellitus | 18716 | 3.2% |
| Coronary artery disease | 6012 | 1.0% |
| Symptoms of COVID-19 (current episode) |  |  |
| Yes | 519503 | 88.8% |

Table S2. Characteristics of 584,846 cases of SARS-CoV-2 infection by period

|  | **Period 1**  **10/01/20 to 12/03/20** | | **Period 2**  **12/04/20 to 04/08/21** | | **Period 3**  **04/09/21 to 06/13/21** | | **Period 4**  **06/14/21 to 08/13/21** | | **Period 5**  **08/14/21 to 10/01/21** | | **Period 6**  **10/02/21 to 12/19/21** | | **Period 7**  **12/20/21 to 03/17/22** | | **Period 8**  **03/18/22 to 05/19/22** | | **Period 9**  **05/20/22 to 08/29/22** | | |
| --- | --- | --- | --- | --- | --- | --- | --- | --- | --- | --- | --- | --- | --- | --- | --- | --- | --- | --- | --- |
|  | N  (45011) | % | N  (86157) | % | N  (26106) | % | N  (14501) | % | N  (9411) | % | N (56310) | % | N (179699) | % | N (81291) | % | N (84156) | % |  |
| Age (years) |  |  |  |  |  |  |  |  |  |  |  |  |  |  |  |  |  |  |  |
| 18 – 29 | 6221 | 13.8% | 12747 | 14.8% | 4193 | 16.1% | 3524 | 24.3% | 1301 | 13.8% | 6926 | 12.3% | 23768 | 13.2% | 5302 | 6.5% | 4990 | 5.9% |  |
| 30 – 39 | 9836 | 21.9% | 20224 | 23.5% | 6756 | 25.9% | 3884 | 26.8% | 2575 | 27.4% | 14048 | 24.9% | 43754 | 24.3% | 14113 | 17.4% | 12954 | 15.4% |  |
| 40 – 49 | 11241 | 25.0% | 22250 | 25.8% | 6759 | 25.9% | 3163 | 21.8% | 2352 | 25.0% | 15358 | 27.3% | 48833 | 27.2% | 19782 | 24.3% | 17919 | 21.3% |  |
| 50 – 59 | 9935 | 22.1% | 17835 | 20.7% | 5311 | 20.3% | 2196 | 15.1% | 1598 | 17.0% | 10595 | 18.8% | 34712 | 19.3% | 19643 | 24.2% | 21898 | 26.0% |  |
| 60 – 69 | 5605 | 12.5% | 9470 | 11.0% | 2387 | 9.1% | 1184 | 8.2% | 1009 | 10.7% | 6952 | 12.3% | 19124 | 10.6% | 14189 | 17.5% | 16957 | 20.1% |  |
| ≥ 70 | 2173 | 4.8% | 3631 | 4.2% | 700 | 2.7% | 550 | 3.8% | 576 | 6.1% | 2431 | 4.3% | 9508 | 5.3% | 8262 | 10.2% | 9438 | 11.2% |  |
| Gender |  |  |  |  |  |  |  |  |  |  |  |  |  |  |  |  |  |  |  |
| Female | 28170 | 62.6% | 53933 | 62.6% | 16645 | 63.8% | 9594 | 66.2% | 6113 | 65.0% | 35614 | 63.2% | 122742 | 68.3% | 56414 | 69.4% | 55686 | 66.2% |  |
| Region of residence |  |  |  |  |  |  |  |  |  |  |  |  |  |  |  |  |  |  |  |
| Île-de-France | 8652 | 19.2% | 21048 | 24.4% | 6838 | 26.2% | 2570 | 17.7% | 1984 | 21.1% | 12972 | 23.0% | 34407 | 19.1% | 18078 | 22.2% | 19489 | 23.2% |  |
| Auvergne-Rhône-Alpes | 10148 | 22.5% | 12666 | 14.7% | 3646 | 14.0% | 1880 | 13.0% | 1219 | 13.0% | 8962 | 15.9% | 25589 | 14.2% | 9484 | 11.7% | 9796 | 11.6% |  |
| Occitanie | 3161 | 7.0% | 6300 | 7.3% | 1926 | 7.4% | 2523 | 17.4% | 1077 | 11.4% | 5361 | 9.5% | 17259 | 9.6% | 7300 | 9.0% | 8789 | 10.4% |  |
| Grand Est | 3718 | 8.3% | 7388 | 8.6% | 1820 | 7.0% | 721 | 5.0% | 643 | 6.8% | 4410 | 7.8% | 18557 | 10.3% | 7140 | 8.8% | 6276 | 7.5% |  |
| Provence-Alpes-Côte d'Azur and Corsica | 3658 | 8.1% | 8741 | 10.1% | 1642 | 6.3% | 2382 | 16.4% | 1542 | 16.4% | 5335 | 9.5% | 14031 | 7.8% | 6169 | 7.6% | 6776 | 8.1% |  |
| Nouvelle-Aquitaine | 2625 | 5.8% | 5059 | 5.9% | 1650 | 6.3% | 1572 | 10.8% | 684 | 7.3% | 4768 | 8.5% | 17339 | 9.6% | 6177 | 7.6% | 7983 | 9.5% |  |
| Hauts-de-France | 4330 | 9.6% | 8721 | 10.1% | 2569 | 9.8% | 696 | 4.8% | 685 | 7.3% | 4234 | 7.5% | 13251 | 7.4% | 7014 | 8.6% | 6275 | 7.5% |  |
| Pays de la Loire | 2094 | 4.7% | 4044 | 4.7% | 1622 | 6.2% | 609 | 4.2% | 391 | 4.2% | 3048 | 5.4% | 10028 | 5.6% | 4524 | 5.6% | 4773 | 5.7% |  |
| Bretagne | 1160 | 2.6% | 2388 | 2.8% | 1069 | 4.1% | 552 | 3.8% | 409 | 4.3% | 2071 | 3.7% | 8898 | 5.0% | 4655 | 5.7% | 4493 | 5.3% |  |
| Bourgogne-Franche-Comté | 2200 | 4.9% | 3547 | 4.1% | 993 | 3.8% | 275 | 1.9% | 285 | 3.0% | 2046 | 3.6% | 7589 | 4.2% | 3403 | 4.2% | 3146 | 3.7% |  |
| Normandie | 1707 | 3.8% | 3446 | 4.0% | 1350 | 5.2% | 478 | 3.3% | 260 | 2.8% | 1579 | 2.8% | 6894 | 3.8% | 3983 | 4.9% | 3303 | 3.9% |  |
| Centre - Val de Loire | 1558 | 3.5% | 2809 | 3.3% | 981 | 3.8% | 243 | 1.7% | 232 | 2.5% | 1524 | 2.7% | 5857 | 3.3% | 3364 | 4.1% | 3057 | 3.6% |  |
| Population of place of residence |  |  |  |  |  |  |  |  |  |  |  |  |  |  |  |  |  |  |  |
| Rural or < 5,000 | 11483 | 25.5% | 21367 | 24.8% | 6482 | 24.8% | 3016 | 20.8% | 1946 | 20.7% | 13301 | 23.6% | 46402 | 25.8% | 20937 | 25.8% | 20786 | 24.7% |  |
| 5,000 - 19,999 | 4441 | 9.9% | 8292 | 9.6% | 2486 | 9.5% | 1315 | 9.1% | 851 | 9.0% | 5301 | 9.4% | 18921 | 10.5% | 8627 | 10.6% | 8773 | 10.4% |  |
| 20,000 - 99,999 | 5513 | 12.2% | 9952 | 11.6% | 2898 | 11.1% | 1671 | 11.5% | 1137 | 12.1% | 6210 | 11.0% | 21308 | 11.9% | 10214 | 12.6% | 10100 | 12.0% |  |
| 100,000 + | 15767 | 35.0% | 27676 | 32.1% | 8137 | 31.2% | 6142 | 42.4% | 3649 | 38.8% | 19573 | 34.8% | 61995 | 34.5% | 25321 | 31.1% | 26964 | 32.0% |  |
| Paris urban area | 7807 | 17.3% | 18870 | 21.9% | 6103 | 23.4% | 2357 | 16.3% | 1828 | 19.4% | 11925 | 21.2% | 31073 | 17.3% | 16192 | 19.9% | 17533 | 20.8% |  |
| Professional category |  |  |  |  |  |  |  |  |  |  |  |  |  |  |  |  |  |  |  |
| Senior executives | 10517 | 35.1% | 24394 | 28.3% | 7423 | 28.4% | 4031 | 27.8% | 2557 | 27.2% | 20244 | 36.0% | 56213 | 31.3% | 24978 | 30.7% | 26274 | 31.1% |  |
| Intermediate profession | 5466 | 18.2% | 15418 | 17.9% | 4495 | 17.2% | 2355 | 16.2% | 1565 | 16.7% | 9412 | 16.7% | 32544 | 18.2% | 13275 | 16.4% | 13151 | 17.2% |  |
| Employees | 5534 | 18.5% | 19861 | 23.1% | 6447 | 24.7% | 3389 | 23.4% | 2105 | 22.4 | 10743 | 19.1% | 38496 | 21.5% | 15773 | 19.4% | 14841 | 20.7% |  |
| Worker/Farmer/ Independent Profession. | 2990 | 9.9% | 9146 | 10.6% | 3203 | 12.3% | 1751 | 12.1% | 1049 | 11.2% | 5014 | 8.9% | 16235 | 9.1% | 5384 | 6.6% | 5621 | 8.9% |  |
| Retired | 4134 | 13.8% | 9148 | 10.6% | 2012 | 7.7% | 1177 | 8.2% | 1168 | 12.4% | 6579 | 11.7% | 20780 | 11.6% | 16797 | 20.7% | 19500 | 14.3% |  |
| Unemployed or inactive | 727 | 2.4% | 3863 | 4.5% | 1183 | 4.5% | 685 | 4.8% | 606 | 6.4% | 2195 | 3.9% | 7431 | 4.1% | 3561 | 4.4% | 3402 | 4.2% |  |
| Student | 626 | 2.1% | 4327 | 5.0% | 1343 | 5.1% | 1113 | 7.8% | 361 | 3.8% | 2123 | 3.8% | 8000 | 4.5% | 1523 | 1.9% | 1367 | 3.7% |  |
| Level of education |  |  |  |  |  |  |  |  |  |  |  |  |  |  |  |  |  |  |  |
| No high school diploma | 1646 | 3.7% | 18366 | 21.3% | 5660 | 21.7% | 2570 | 17.7% | 2027 | 21.5% | 9067 | 16.1% | 30727 | 17.1% | 14204 | 17.5% | 14823 | 17.6% |  |
| High school diploma | 1538 | 3.4% | 18290 | 21.2% | 5584 | 21.4% | 3098 | 21.4% | 1934 | 20.6% | 9958 | 17.7% | 34533 | 19.2% | 15407 | 19.0% | 15432 | 18.3% |  |
| Bachelor's degree | 2409 | 5.4% | 29624 | 34.4% | 8905 | 34.1% | 5087 | 35.1% | 3199 | 34.0% | 19742 | 35.1% | 65416 | 36.4% | 30117 | 37.0% | 31083 | 36.9% |  |
| Master's degree | 39418 | 87.6% | 19877 | 23.1% | 5957 | 22.8% | 3746 | 25.8% | 2251 | 23.9% | 17543 | 31.2% | 49023 | 27.3% | 21563 | 26.5% | 22818 | 27.1% |  |
| [Comorbidities](https://context.reverso.net/traduction/anglais-francais/comorbidities) |  |  |  |  |  |  |  |  |  |  |  |  |  |  |  |  |  |  |  |
| Obesity | 7276 | 16.2% | 14049 | 16.3% | 4362 | 16.7% | 1740 | 12.0% | 1324 | 14.1% | 7433 | 13.2% | 27066 | 15.1% | 14214 | 17.5% | 14993 | 17.8% |  |
| Hypertension | 4323 | 9.6% | 7678 | 8.9% | 2045 | 7.8% | 920 | 6.3% | 766 | 8.1% | 4628 | 8.2% | 15692 | 8.7% | 11009 | 13.5% | 12312 | 14.6% |  |
| Chronic respiratory disease | 3787 | 8.4% | 6701 | 7.8% | 2097 | 8.0% | 1155 | 8.0% | 789 | 8.4% | 4033 | 7.2% | 14976 | 8.3% | 7232 | 8.9% | 7484 | 8.9% |  |
| Immunosuppression | 1211 | 2.7% | 2278 | 2.6% | 689 | 2.6% | 375 | 2.6% | 287 | 3.0% | 400 | 0.7% | 7015 | 3.9% | 4421 | 5.4% | 4666 | 5.5% |  |
| Diabetes mellitus | 1512 | 3.4% | 2583 | 3.0% | 690 | 2.6% | 329 | 2.3% | 281 | 3.0% | 1383 | 2.5% | 4916 | 2.7% | 3251 | 4.0% | 3620 | 4.3% |  |
| Coronary artery disease | 471 | 1.0% | 759 | 0.9% | 141 | 0.5% | 105 | 0.7% | 77 | 0.8% | 477 | 0.8% | 1593 | 0.9% | 1084 | 1.3% | 1260 | 1.5% |  |
| Symptoms of COVID-19 (current episode)^a^ |  |  |  |  |  |  |  |  |  |  |  |  |  |  |  |  |  |  |  |
| Yes | 30676 | 68.2% | 73757 | 85.6% | 22564 | 86.4% | 13190 | 91.0% | 8431 | 89.6% | 51858 | 92.1% | 161175 | 89.7% | 76197 | 93.7% | 80327 | 95.5% |  |
| History of SARS-CoV-2 infection^b^ |  |  |  |  |  |  |  |  |  |  |  |  |  |  |  |  |  |  |  |
| Yes | / | / | / | / | 645 | 2.5% | 554 | 3.8% | 340 | 3.6% | 1961 | 3.5% | 13233 | 7.4% | 6076 | 7.5% | 13232 | 15.7% |  |

^a^ Collection of data concerning the symptomatic status of ongoing infections was introduced from period 2 onward. In period 1, participants were categorized as symptomatic only if their symptoms were the primary reason for the test.

^b^ Until February 18, 2021, participants with a history of SARS-CoV-2 infection (virological or serological test) were not included in the study.

Table S3. Characteristics of the source cases according to their origin between October 1, 2020, and August 29, 2022

|  | Household  (n = 125 397) | | Work  (n = 35 627) | | Family  (n = 46 132) | | Friend  (n = 26 580) | | *P* value |
| --- | --- | --- | --- | --- | --- | --- | --- | --- | --- |
| Age (years) Median (IQR) | 28 (11-48) | | 40 (30-50) | | 40 (26-60) | | 39 (28-55) | |  |
|  |  |  |  |  |  |  |  |  |  |
|  | N | % | N | % | N | % | N | % |  |
| Age category |  |  |  |  |  |  |  |  | *< 0.001* |
| < 18 years | 48115 | 38.4% | 2063 | 5.8% | 6296 | 13.6% | 1277 | 4.8% |  |
| 18-29 years | 16308 | 13.0% | 5085 | 14.3% | 7814 | 16.9% | 6347 | 23.8% |  |
| 30-49 years | 32544 | 26.0% | 17079 | 47.9% | 13842 | 30.0% | 10028 | 37.7% |  |
| 50-69 years | 24201 | 19.3% | 10417 | 29.2% | 11893 | 25.8% | 6611 | 24.8% |  |
| ≥ 70 years | 4229 | 3.4% | 983 | 2.8% | 6287 | 13.6% | 2317 | 8.7% |  |
| Gender |  |  |  |  |  |  |  |  | *< 0.001* |
| Female | 51128 | 40.8% | 17787 | 49.9% | 25592 | 55.5% | 14381 | 54.1% |  |
|  |  |  |  |  |  |  |  |  |  |

Table S4. Characteristics of single-encounter meetings during which SARS-CoV-2 infection took place according to the origin of the source case (household members excluded) and stratified by the symptomatic status of the source case between October 1, 2020 and August 29, 2022

|  | **Total** | | | | **P value** | **Work** | | | | **P value** | **Family** | | | | **P value** | **Friends** | | | | **P value** |
| --- | --- | --- | --- | --- | --- | --- | --- | --- | --- | --- | --- | --- | --- | --- | --- | --- | --- | --- | --- | --- |
|  | **Symptomatic** | | | |  | **Symptomatic** | | | |  | **Symptomatic** | | | |  | **Symptomatic** | | | |  |
|  | **No** | | **Yes** | |  | **No** | | **Yes** | |  | **No** | | **Yes** | |  | **No** | | **Yes** | |  |
|  | N | % | N | % |  | N | % | N | % |  | N | % | N | % |  | N | % | N | % |  |
|  | 45259 | 64.8% | 24529 | 35.1% |  | 6798 | 55.9% | 5366 | 44.1% |  | 13289 | 61.3% | 8379 | 38.7% |  | 13094 | 72.1% | 5070 | 27.9% |  |
| Location of encounter |  |  |  |  | <0.001 |  |  |  |  | < 0.001 |  |  |  |  | 0.020 |  |  |  |  | 0.316 |
| Indoors with closed windows | 31873 | 70.4% | 17357 | 70.8% |  | 5176 | 76.1% | 4147 | 77.3% |  | 9488 | 71.4% | 5835 | 69.6% |  | 9040 | 69.0% | 3472 | 68.5% |  |
| Indoors with open windows | 9436 | 20.9% | 5251 | 21.4% |  | 1253 | 18.4% | 1006 | 18.7% |  | 3050 | 23.0% | 2031 | 24.2% |  | 3040 | 23.2% | 1171 | 23.1% |  |
| Outdoors | 3944 | 8.7% | 1919 | 7.8% |  | 369 | 5.4% | 213 | 4.0% |  | 751 | 5.7% | 513 | 6.1% |  | 1014 | 7.7% | 427 | 8.4% |  |
| Duration of encounter |  |  |  |  | <0.001 |  |  |  |  | 0.002 |  |  |  |  | < 0.001 |  |  |  |  | 0.346 |
| Less than a minute | 3831 | 8.5% | 2519 | 10.3% |  | 826 | 12.2% | 702 | 13.1% |  | 1046 | 7.9% | 839 | 10.0% |  | 979 | 7.5% | 410 | 8.1% |  |
| Less than 5 minutes | 5441 | 12.0% | 3247 | 13.2% |  | 1103 | 16.2% | 970 | 18.1% |  | 1457 | 11.0% | 1006 | 12.0% |  | 1312 | 10.0% | 537 | 10.6% |  |
| 5-15 minutes | 6238 | 13.8% | 3356 | 13.7% |  | 1187 | 17.5% | 953 | 17.8% |  | 1657 | 12.5% | 1024 | 12.2% |  | 1477 | 11.3% | 572 | 11.3% |  |
| More than 15 minutes | 23277 | 51.4% | 11929 | 48.6% |  | 2790 | 41.0% | 2019 | 37.6% |  | 6889 | 51.8% | 4089 | 48.8% |  | 7536 | 57.6% | 2894 | 57.1% |  |
| Unknown | 6467 | 14.3% | 3476 | 14.2% |  | 892 | 13.1% | 722 | 13.5% |  | 2240 | 16.9% | 1421 | 17.0% |  | 1790 | 13.7% | 657 | 13.0% |  |
| Mask-wearing^a^ |  |  |  |  | <0.001 |  |  |  |  | < 0.001 |  |  |  |  | < 0.001 |  |  |  |  | < 0.001 |
| Neither wore a mask | 38568 | 85.2% | 18762 | 76.5% |  | 4262 | 62.7% | 2907 | 54.2% |  | 12479 | 93.9% | 7272 | 86.8% |  | 12646 | 96.6% | 4718 | 93.1% |  |
| By suspected source of infection only | 968 | 2.1% | 1120 | 4.6% |  | 215 | 3.2% | 328 | 6.1% |  | 217 | 1.6% | 381 | 4.5% |  | 101 | 0.8% | 126 | 2.5% |  |
| By person infected only | 2223 | 4.9% | 2000 | 8.2% |  | 772 | 11.4% | 800 | 14.9% |  | 293 | 2.2% | 308 | 3.7% |  | 156 | 1.2% | 116 | 2.3% |  |
| By both source case and participant | 3494 | 7.7% | 2645 | 10.8% |  | 1549 | 22.8% | 1331 | 24.8% |  | 300 | 2.3% | 418 | 5.0% |  | 191 | 1.5% | 110 | 2.2% |  |
| **Total** | 45254 |  | 24527 |  |  | 6798 |  | 5366 |  |  | 13289 |  | 8379 |  |  | 13094 |  | 5070 |  |  |

^a^ Mask-wearing during the last encounter with the source when multiple encounters occurred.

Table S5. Characteristics of the suspected events (single suspicion) at the origin of the infection for participants without an identified source case between October 1, 2020, and August 29, 2022

| Source of infection for suspected events (N = 132 401)^a^ | N | (%) |
| --- | --- | --- |
| Setting of event |  |  |
| Work-related | 25416 | 27.2% |
| Friends | 18130 | 19.4% |
| Family | 17491 | 18.8% |
| Cultural/Entertainment | 5984 | 6.4% |
| Sports | 4757 | 5.1% |
| Religious | 980 | 1.1% |
| Other | 20506 | 22.0% |
| Type of event |  |  |
| Shared meal | 16964 | 18.2% |
| Meeting | 9793 | 10.5% |
| Party | 7312 | 7.8% |
| Public transportation | 6836 | 7.3% |
| Show, entertainment venue | 5316 | 5.7% |
| Visit to health care facility | 5041 | 5.4% |
| Ceremony (wedding, baptism, graduation etc.) | 3129 | 3.4% |
| Drink or coffee | 2195 | 2.4% |
| Sports club/wellness area | 1202 | 1.3% |
| Other event | 35476 | 38.0% |
| Reason for suspicion |  |  |
| No physical distancing | 45413 | 48.7% |
| None wore a mask | 15388 | 16.5% |
| Large number of people | 11558 | 12.4% |
| Other | 20905 | 22.4% |

^a^ Suspicion of several events are excluded of the table n= 39137

Figure S1. Presentation of the nine study periods according to government measures, epidemic waves, and variants between October 1, 2020, and August 29, 2022 (data on the incidence rate of SARS-CoV-2 infection and the prevalence of circulating strains were obtained from public data provided by *Santé publique France*)


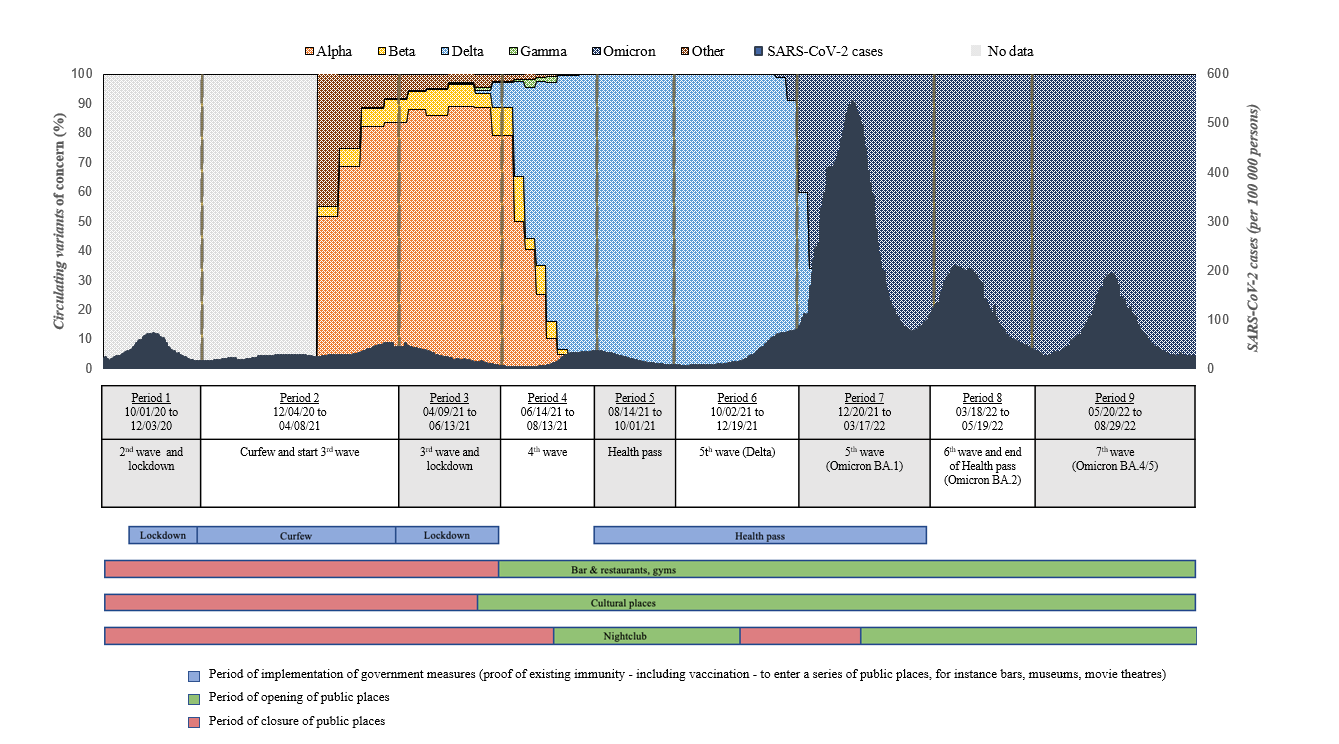


Figure S2. Proportion of encounters for which the source case was symptomatic in the single encounter that caused infection according to the setting of the meeting by period


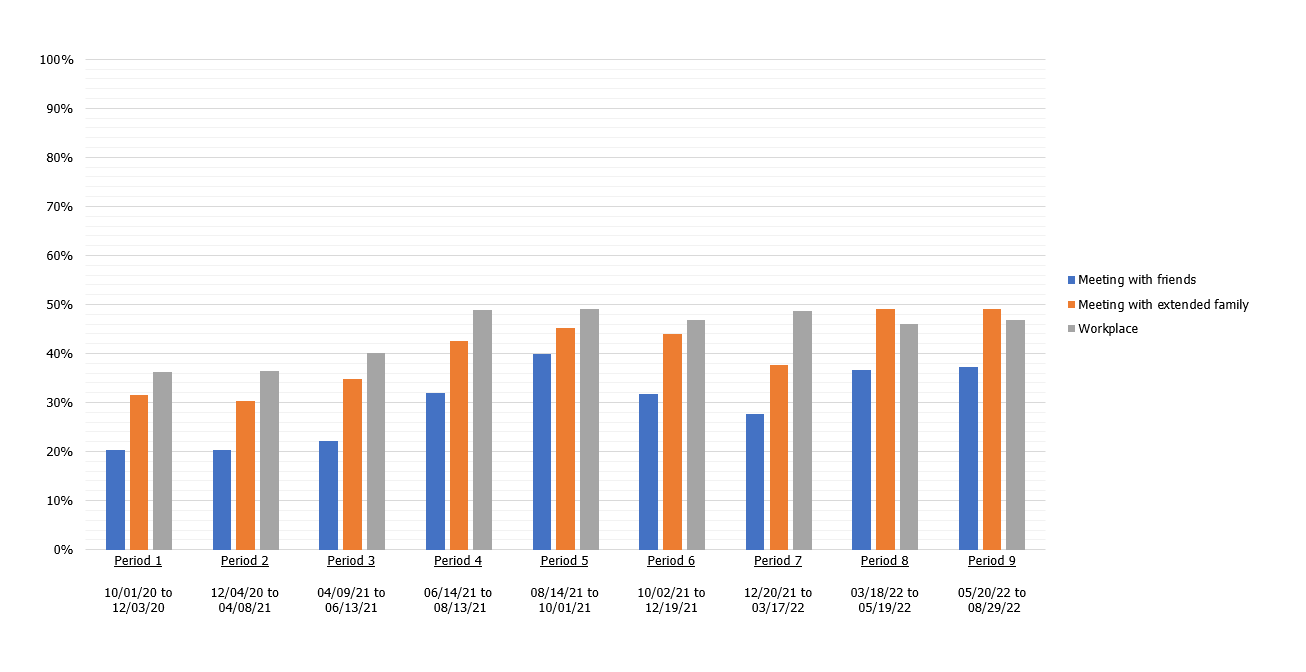


Figure S3. Proportion of encounters for which neither the source case nor the participants wore a mask during the single encounter that caused the infection according to the setting of the meeting by period


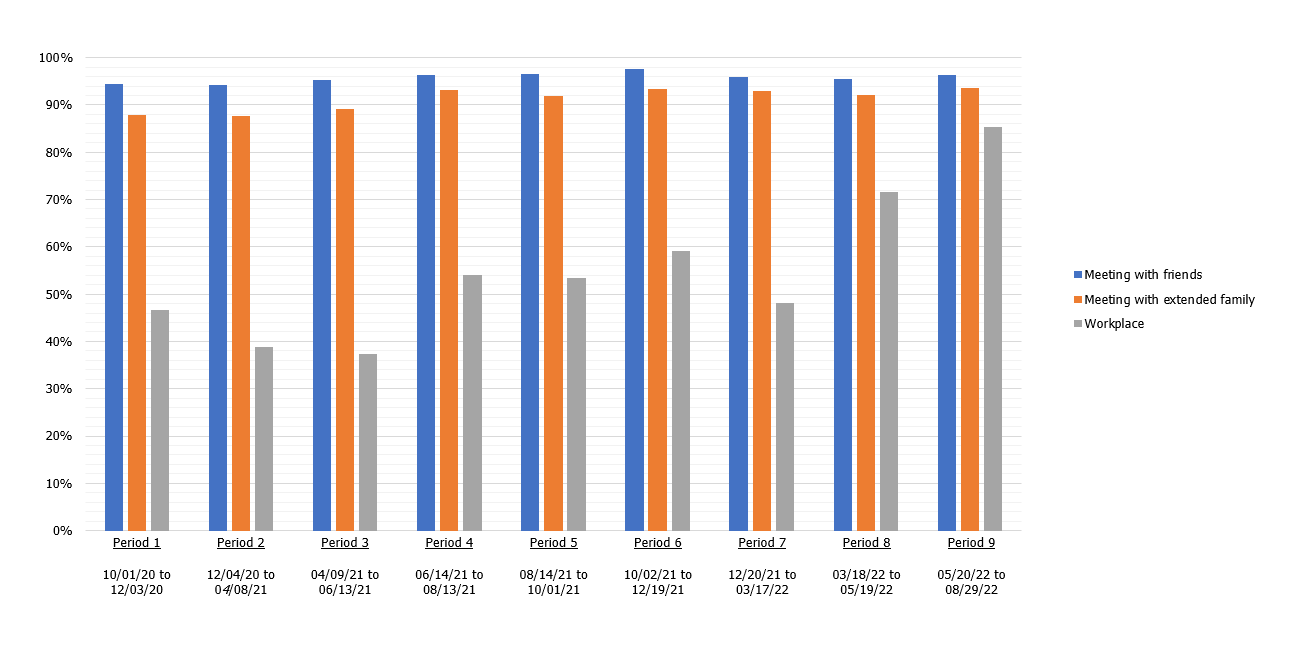

Supplement: Supplementary file 1 — Additional file 1. [file 12889_2024_17772_MOESM1_ESM.docx]
